# The Oncohumanities training programme: Fostering a deeper engagement and integration of oncology and humanities to tackle the pressing and complex challenges of cancer care

**DOI:** 10.1177/03008916231158674

**Published:** 2023-03-09

**Authors:** Daniele Carrieri, Fedro Alessandro Peccatori, Giovanni Boniolo

**Affiliations:** 1 University of Exeter Medical School, University of Exeter, Exeter, UK; 2 European Institute of Oncology IRCCS, Milan, Italy; 3 Department of Neuroscience and Rehabilitation, University of Ferrara, Ferrara, Emilia-Romagna, Italy

**Keywords:** Oncology care, patient centred care, oncology training

## Abstract

‘Oncohumanities’ is a new field of oncology and humanities which integrates a rich gamut of humanity disciplines and oncological expertise to tackle patients’ real needs and priorities. To promote knowledge and awareness on this topic, we propose a training programme that will blend conceptual knowledge underpinning oncology practice with and person-centred care based on the humanisations of care, on empowerment of patients, and on respect for their diversities. Oncohumanities differs from most existing medical humanities training as it is integrated and engaged with oncology (rather than an add-on). This means that its agenda is driven by the real needs and priorities arising out of daily oncological practice. It is our hope that this new Oncohumanities programme and approach will contribute to guiding future efforts to foster a strong integrated partnership between humanities and oncology.

## Introduction

Cancer continues to be one of the most pressing and complex global health problems. Cancer can involve the entire health system and the full life course of individuals. It is the second leading cause of death globally,^a^ and is linked to significant health inequalities within and across countries.^1-3^ For example, in high income countries 90% of children are likely to access a cure, while in the developing countries only 10% have a chance of survival.^1,4^ Even in high income countries there are inequities in access to cancer care – which are linked to late diagnosis, but also overdiagnosis and overtreatment.^4^ The ongoing disruptions caused by the COVID-19 pandemic are further exacerbating these disparities in access to cancer screening and care, increasing excess mortality.^5^

Given the prevalence and complexity of cancer, optimising cancer care has become a key priority which includes other health systems challenges such as prevention and palliation.^5^ Moreover, patient quality of life and more holistic and person-centred approaches are increasingly becoming a hallmark of cancer care.^6^ Therefore, setting high standards for equitable and excellent cancer care can also work as a catalyst for the optimisation of a biopsychosocial model of medicine^7^ – and a more patient-centred healthcare service provision.^4^

Addressing these complex and far-reaching cancer challenges requires multipronged strategies and a truly multidisciplinary approach, within and beyond cancer care and medicine.^4,8^ Multidisciplinary training and research in oncology represent an important foundation of such a multipronged strategy.

The European School of Oncology (ESO) – an independently funded non-profit organisation dedicated to quality training and providing oncology education to help the improvement of cancer patients’ treatment – has developed an innovative educational postgraduate programme in Oncohumanities (to be launched in 2023). At the core of this training is the new term ‘Oncohumanities’, defined as the set of deep reflections on conceptual, sociological, anthropological, and ethical issues, that allow the integration of technical aspects of cancer treatment with what is necessary to render them humane on the basis of an understanding of patients’ multilevel life course experience of cancer, and the respect of their autonomy, dignity, and of their life-style, values and beliefs. The programme enables oncologists to advance individual competencies and strategies to bridge the gap between daily practices and humanistic reflections on these practices, for the benefits of the patients.

## The Oncohumanities training programme

The Oncohumanities training programme provides state-of-the-art best knowledge on conceptual, sociological, anthropological, and ethical debates focusing on research, decision-making and diagnosis, treatment options and planning of oncology care. The main objective of the programme is to improve knowledge underpinning oncology practice, and to enhance the oncology practitioner’s sensitivity to person-centred care attitudes based on the humanisation of care, on empowerment of the patients, and on respect for their diversities. To the best of our knowledge no continuing education programmes in Oncohumanities – understood in this broad meaning – are currently offered.

This unique programme, albeit developed for oncologists, is also open to any healthcare practitioners, and to non-hospital personnel interested in humanities-related issues in oncological settings. The programme will include:

- Critical, deterministic and probabilistic reasoning in clinical settings
- Conceptual, sociological, anthropological, and ethical aspects of oncological screening and testing, and on the issues of overdiagnosis and overtreatment
- Conceptual, sociological, anthropological, and ethical aspects related to patients’ spiritual, cultural, and gender diversity
- Conceptual, sociological, anthropological, and ethical aspects dealing with end of life, supportive care and palliation
- Conceptual, sociological, anthropological, and ethical aspects dealing with paediatric, aging, oncofertility, and survivorship issues
- Conceptual, sociological, anthropological, and ethical aspects dealing with the relations between the oncologists, the nurses and the patients and their relatives.

## The novelty of this training programme: The Oncohumanities approach

The fundamental novelty of the Oncohumanities programme lies in its methodological approach: it is integrated to and engaged with the medical practice of oncology; and it draws on a wide range of humanities disciplines.

This approach is radically different from most past and existing humanities training in oncology (and general medical humanities)^9^ – e.g. narrative medicine or art and medicine approaches – which tend to be ‘add-ons’ to oncology (and medicine), and may not always be of relevance to, or up to date with, the real daily clinical challenges and needs arising from oncology practice. The Oncohumanities programme aims to provide oncology healthcare practitioners with the competencies to better understand the human implications of oncology care; that is to better understand the needs of the patient beyond the strictly biomedical aspects of oncology care.

**Figure 1.**
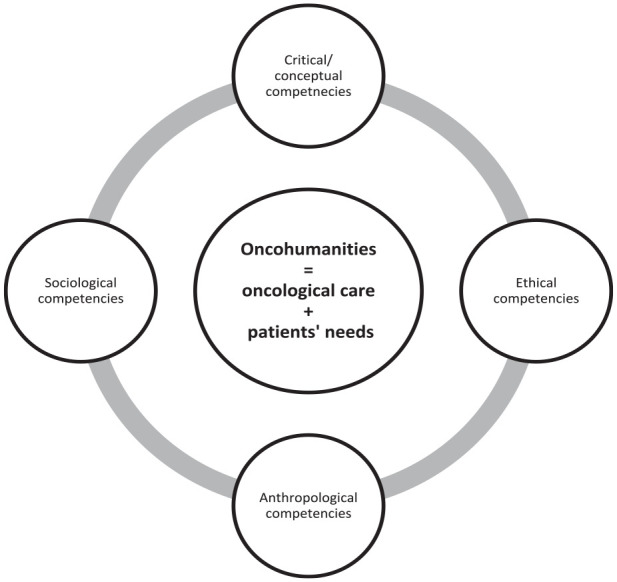
Oncohumanities is integrated to oncology practice and provides tools and competencies for oncologists to better understand the human implications of oncology care.

Therefore Oncohumanities promotes a novel understanding of humanities which includes subjects like philosophy of science, anthropology, ethics and sociology; and considers humanities as being integral to the core oncological curriculum itself; or, in other words, to the key competences of a good oncology healthcare practitioner (see Figure 1).

Although the Oncohumanities programme is novel in the field of oncology, there are similar examples of strong integration of humanities with different medical and scientific disciplines. Recent examples can be found in relation to science and philosophy;^10^ neuroscience and humanities (‘neurohumanities’);^11^ ethical counselling in genomic medicine;^12^ as well as the development of real training models which introduce humanistic studies to a scientific environment.^13^

This new Oncohumanities approach - by fostering a strong integrated partnership between humanities and oncology - will benefit oncology training and practice, and ultimately patients. We outline the main features of this integrated and engaged approach below (for an in depth discussion of this see Boniolo et al.)^14^

### Integration

The integrative facet of the Oncohumanities approach means that its agenda is driven by the real needs and priorities arising out of daily oncological practice. Such integration of a rich gamut of humanity disciplines to oncology not only provides a strong platform to foster truly patient-centred care. It also strengthens oncology practitioners’ understanding of their own specialty, and how it relates to other medical specialties. Oncohumanities can also foster a much-needed conceptual understanding of the technological, scientific, and clinical aspects of oncological practice - including overdiagnosis, overtreatment, inequities, sustainability of cancer care;^15^ understanding of cancer probability and screening;^16,17^ clinical ethics and decision making;^12^ communication skills; and clinical laws and regulations within and across countries.^18^

### Engagement

The engaged facet of the Oncohumanities approach means that Oncohumanities is a platform for a truly open dialogue between oncological practice, training and research, and the humanities broadly conceived.

In line with this approach, the Oncohumanities training has been co-developed with key stakeholder which include ESO; the Department of Philosophical and Communication Studies, supported by the Medical Oncology Unit – Department of Experimental, Diagnostic and Specialty Medicine- University of Bologna; and internationally recognised sociologists, philosophers, anthropologists and ethics scholars working in the field of oncology. To inform the development of the Oncohumanities programme, ESO is also conducting an anonymous survey administered to oncology practitioners and healthcare professionals in other specialties, to understand their interest in conceptual, sociological, anthropological, and ethical aspects in relation to many areas related to oncology (from aging to survivorship).^b^

### Evaluation

For both the short- and long-term success of Oncohumanities it is also important to regularly evaluate and review (using surveys and other tools) the training, to assess its impact on oncology practitioners’ sense of preparedness, fulfilment, and on clinical and research practice in oncology for example. This evaluation and review activity can be seen as part of the engagement facet of the Oncohumanities approach. As often happens with training programmes and other complex initiatives, it can be challenging to find adequate ways to measure success and impact. There have been some attempts in the medical humanities arena,^16,17^ although it is difficult to find adequate indicators to measure success. Enjoyment from work, resource gains, and shifts in data indicators (from quantitative to qualitative) could provide useful measures.^18^

## Notes

a. WHO Cancer https://www.who.int/health-topics/cancer#tab=tab_1

b. The link to the survey can be found here https://docs.google.com/forms/d/e/1FAIpQLSe6qqqJLEaK1687s-E-_Jzk93mF4t_73RnJzWZrvtBmLa9AtA/viewform
